# Loneliness: An Existential and Public Health Issue

**DOI:** 10.3390/ijerph22101544

**Published:** 2025-10-09

**Authors:** Ami Rokach, David Berman

**Affiliations:** Department of Psychology, Faculty of Health, York University, Toronto, ON M3J 1P3, Canada; davideb.htc@yorku.ca

## 1. Loneliness, a Universal Issue

Interpersonal relationships are a fundamental human need [1]. Indeed, having strong social and personal relationships contributes to multiple forms of physical and mental health and well-being [2,3]. Loneliness is part of life. Whether frequently or rarely, all humans know the deep and passing pangs of loneliness, of feeling dejected, unimportant, forgotten, and alone [4]. The results of recent studies have revealed an increase in the number of people who state that they have nobody to confide in, demonstrating a significant loss of social contacts compared to past generations [5]. Loneliness may be reactive, when it is experienced as a reaction to a life event, or essential, when it is interwoven into one’s personality [6]. There are several variables that are associated with loneliness, such as living alone, being unmarried, not participating in social groups, and having fewer friends to turn to in times of need [7]. Additionally, lack of social support, be it from a close family or friends, may further exacerbate the experience of loneliness [8].

McCullough [9] observed that when one does not feel related to others, feels that one does not matter to them, the feeling of loneliness tends to increase. In the study by A. Flett et al. [10], the authors examined the relationship between belief in mattering to others and loneliness in a sample of 232 undergraduate students. Their results revealed a robust and significant correlation between feeling that one does not matter to others and experiencing loneliness.

Although some may be more vulnerable to the effects of loneliness, it would appear that all are bound to experience this feeling [11]. But who are the lonely? In a study in which lonely people were asked about their experiences, responses such as feeling detached, distanced, and isolated were highlighted. The lonely reported that they feel unimportant, that they do not matter to others, that they are, sometimes, even unnoticed by those around them, and that while they may be surrounded by caring people, they *feel* that no one cares [6].

In addition, the public health implications of loneliness are considerable, given its far-reaching impact on both quality of life and overall health. The results of numerous studies have established clear links between loneliness and adverse health outcomes, including worse cardiovascular and cognitive health [12], a risk factor for dementia and Alzheimer’s disease [13], greater likelihood of stroke [14], increased risk of depression [15], and elevated mortality [16].

The present Special Issue includes a variety of articles and studies, the results of which highlight the richness of the experience of loneliness and capture its effects across different age groups and life journeys. This Special Issue is divided into five conceptual sections:

## 2. Part 1: Defining and Assessing Loneliness

Mills et al. (Contribution 1) focused, in their study, on loneliness in adults with opioid use disorder. They highlighted the psychological correlates of loneliness and its effects on those struggling with addiction.

Rokach et al. (Contribution 2) describe, in their article, the development and validation of a scale aimed at assessing how loneliness is expressed and experienced in intimate relationships. They review the Loneliness in Intimate Relationships Scale (LIRS) in their article.

## 3. Part 2: Loneliness and Physical and Mental Disorders

Vallee (Contribution 3) highlights the association between loneliness and cardiac problems. Indeed, as the current literature indicates, in general, a positive correlation between loneliness and atherosclerotic cardiovascular disease was found.

*Einav & Margalit* (Contribution 4) investigated the feeling of loneliness before and after the COVID-19 pandemic, as many individuals experienced loneliness and social alienation during this particular period. They found lower levels of loneliness and a sense of coherence among those questioned before the COVID-19 pandemic compared to those measured after the pandemic.

Patrono et al. (Contribution 5) also sought to determine the impact that COVID-19 had on young adults living in Italy. They found an alarming positive correlation between the length of experienced loneliness and youth rule-breaking behaviour.

Goldberg et al. (Contribution 6) address the isolation and related stigma of family members who live with and care for those with mental health problems. Their results indicated that those living with a mentally ill relative experienced comparatively higher levels of stigma by association compared with those who did not live with such a relative. The authors reported that both groups experienced moderate levels of loneliness and noted that the cohabiting relatives perceived themselves as lacking support from friends and other family members.

## 4. Part 3: Loneliness in Various Age Groups

Wright & Silard (Contribution 7) examined loneliness in young adult workers in Western Europe. Their results indicated that workers feel invisible at work, have a thwarted sense of belonging to their employers, and consequently experience regular relational deficiencies due to automation and individualisation of work practices.

Ezeokonkwo et al. (Contribution 8) focused their study on older adults and examined the relationship between their interpersonal goals and loneliness. Their results showed that interpersonal goals were significantly negatively associated with loneliness. In other words, those with higher compassion and self-image goals experienced less loneliness.

Ramírez López (Contribution 9) examined the role of functional deficits, depression, and cognitive symptoms in Mexican older adults on their perceived loneliness. Loneliness was found to be associated with depression and low levels of instrumental activities of daily living but not with cognitive impairment.

## 5. Part 4: Loneliness and Destructive Behaviours

Herczyk et al. (Contribution 10) focused on a sample of individuals with substance abuse disorder and explored the association between loneliness and mindfulness in this particular population. They found that rates of loneliness, depression, and anxiety did not differ between those who continued treatment and those who did not. They noted that rates of mindfulness, which was associated with effective treatment in preliminary findings, were significantly lower among those not retained versus those retained.

Rokach & Chan (Contribution 11) focus on love and the devastating effects of infidelity. It is acknowledged that infidelity, and its negative effects, is relatively prevalent among Western societies. In this particular study, infidelity was associated with loneliness, which may be the cause as well as the result of infidelity, in addition to the association between infidelity and stress and heartache. Infidelity can damage a loving, romantic relationship to the point of its demise.

Yong, R.K.F. (Contribution 12) explored the effects of unemployment and living alone on loneliness and suicidality in Japan. Their results indicated that unemployment and living alone each elevated the risk of suicidality, with the highest rate among unemployed men aged 40–59 living alone. Among women, interaction was most evident for those aged 40–59 and 25 and sub-additive at ≥60.

## 6. Part 5: Addressing Loneliness

Matthaeus et al. (Contribution 13) conducted a study on online dyadic socio-emotional vs. mindfulness-based training. They found that a mental training approach, based on a 12-min novel online practice routine conducted with a partner, was quite effective in reducing pain and could potentially help address the increasing problem of loneliness in our society.

Bjørnøy Urke et al. (Contribution 14) compared the efficacy of a single-tiered vs. multi-tiered approach at preventing loneliness in students from upper secondary schools in Norway. The results highlighted that a multi-tier intervention reduced the feeling of loneliness in the second year of upper secondary school and did so by utilizing a caring school approach during the students' first year. In contrast, the single-tier intervention was associated with increased loneliness due to a decrease in the perception of a caring school climate.

Johnsrud et al. (Contribution 15) sought to explore the effect of outdoor activities on an individual’s health and loneliness. They examined the impact of garden installations at inexpensive summer cottages, where dwellers tended and cared for various plants. They found that maintaining such a garden positively impacts self-perceived well-being and physical health through exercise and outdoor activities. They also noted that the allotment garden, which individuals tended to, strongly impacts perceived health, well-being, and sense of coherence for the individuals, by promoting outdoor activities and social interaction while preventing feelings of loneliness and isolation.

Cattaneo et al. (Contribution 16) explored how Nature-Based Social Prescription (NBSPs)-guided group activities could assist people experiencing social isolation and loneliness. They uncovered a holistic health paradigm linking nature, community, and well-being, in addition to stark ecological inequities with limited green-space access in deprived districts, and discovered work challenges that rose as a result of the urgent needs of individuals facing significant socio-economic challenges in demanding contexts. NBSP appears to be a promising approach to addressing loneliness.

Dwyer (Contribution 17) views digital interventions, such as artificial intelligence companions, as methods for fostering connection and mitigating individual negative experiences of loneliness. Based on communication studies and behavioural information design, the author found that loneliness is understood both as an emotional or interpersonal state and as a logical consequence of hegemonic digital and technological design paradigms. The author proposes a model for evaluating and designing digital public health interventions that resist behavioural enclosure and support autonomy, relational depth, systemic accountability, and structural transparency.

                   * *

The impact of loneliness can be devastating and long-lasting. Mijuskovic [17] poignantly described our social milieu as becoming increasingly contaminated and ironically claimed separation is becoming one of the only things we share. Although responsibility and commitment toward one another are the true salvation of our communities, collaboration has dwindled and been replaced by competition (see also Surkalim et al., [18]).

While these structural causes of loneliness are persistent, the experience of loneliness can be brief, where bouts of the experience have been termed transient loneliness. Loneliness may be experienced occasionally and may not be associated with long-term negative effects [19]. However, experiencing loneliness on a regular or chronic basis may induce a host of emotional, behavioural, and cognitive implications [20,21].

Through this Special Issue, we have not only attempted to highlight the destructive effects of loneliness but also indicate that loneliness need not be a constant companion. Loneliness can, indeed, be addressed, controlled, and need not rule our lives. It has been repeatedly demonstrated that the first step in addressing loneliness is what we have attempted to achieve herein: admit—to ourselves—that we are lonely, recognize and address the learned helplessness which prevents many individuals from actively improving their lot in life, explore the reasons that we experience such isolation, and earnestly attempt to control the loneliness we experience.

### Concluding Comments

This Special Issue highlights the multifaceted nature of loneliness, its profound impact on individuals and communities, and what its alarming rise reveals about our present age. To this end, this Special Issue includes diverse investigations into the causes and correlates of loneliness amidst persistent political, technological, economic, and global health challenges. These studies include works that underscore the troubling links between loneliness and issues such as anxiety, depression, and substance abuse (Contributions 1 and 10), all of which appear to be intractable issues plaguing Western societies.

This Special Issue also explores innovative approaches to understanding loneliness within intimate relationships. While such relationships are often viewed as a safeguard against loneliness, this research highlights how they can instead contribute to this issue, particularly when confronted with conflict and infidelity (Contribution 11). Developments in this area of study are further supported by the introduction of a new Loneliness in Intimate Relationships Scale (Contribution 2).

Other contributions examine the impact of the COVID-19 pandemic, wherein the social isolation experienced during lockdowns was linked to heightened levels of loneliness thereafter (Contribution 4). Additionally, this research shows that male youths who lived through the lockdowns exhibited increased rates of rule-breaking behaviours (Contribution 5). While extensive research will be required to fully contextualize the long-term psychological effects of this global disruption on millions of individuals, it is already clear that societies will be grappling with its consequences for many years to come.

Addressing the complex nature of loneliness, this Special Issue also examines its associations with both physical and psychological disorders, whereby Vallée (Contribution 3) draws attention to how loneliness is a risk factor for cardiovascular disease in men, while Goldberg et al. (Contribution 6) explore the stigmatization faced not only by individuals with mental illness but also by their families. This stigma is linked to a lack of compassion, relationships, and understanding and elevated levels of loneliness.

In the included articles, loneliness was acknowledged as a pervasive issue across all age groups. Younger workers, for instance, often experience loneliness during work hours, struggling to build meaningful connections with coworkers in modern workplaces that prioritize competition over collaboration (Contribution 7). Similarly, Urke et al. (Contribution 14) investigated various approaches to reduce student loneliness. In contrast, López et al. (Contribution 9) and Ezeokonkwo et al. (Contribution 8) examined the close relationship between loneliness and older adults, highlighting how a decline in interpersonal goals and instrumental activities can leave individuals increasingly vulnerable in later life.

These research efforts also included practical approaches to understanding and addressing loneliness, such as how mindfulness can help mitigate substance abuse disorder and loneliness (Contribution 10). Similarly, Urke et al. (Contribution 14) showed that a multi-tier intervention of mindfulness effectively reduced loneliness in students. Another study in this Special Issue examined the positive impact of gardening on loneliness, showing how an improved sense of coherence and increased physical activity can provide meaningful benefits (Contribution 15).

The 17 included papers reveal the complexities surrounding the manifestation and experience of loneliness. They underscore the need for remediation efforts that extend beyond individual-level interventions to include social and workplace considerations. The apparent surge in loneliness in modern life has been linked to a raft of social issues, including the lack of community [7], the decline of “third spaces” [22], and the pervasive influence of social media and related technologies [23]. Addressing these challenges will require a herculean effort that includes collective and community-driven contributions.

With the inevitability of future global pandemics and growing concerns about bird flu [24,25], it is crucial to prioritize efforts to understand and mitigate loneliness, particularly in the context of the reintroduction of government-mandated social restrictions. It is also worth reevaluating the heavy reliance on technological solutions for combating loneliness and fostering sociability. Such approaches have often proven to be of limited efficacy and, in some instances, may have exacerbated the problem [26,27]. The need to investigate the associated harms of these technologies is only made more pressing with the introduction of AI tools and AI-integrated humanoid robotics to explicitly provide “companionship” and address loneliness [28]. Much like social media, governments appear intent on allowing these technologies to proliferate without any serious consideration of the harm they might cause. Because of these existing concerns and developments, emphasizing authentic human interactions and community-building efforts seems more important than ever. While these technologies hold the promise of keeping people connected, especially during times of government-mandated social restrictions, they should be integrated into methods that enhance and support existing social connections and practices rather than allowing them to further encroach on how we interact and connect with one another.

Despite the above concerns, research efforts like those presented in this Special issue offer hope—a powerful confound to loneliness—along with the connections fostered as academics unite to address this pressing issue. These efforts can inform policy approaches that emphasize public and professional education programs aimed at raising awareness and reducing stigma, while ensuring that the negative health consequences of loneliness are not only recognized but addressed through innovative prevention strategies, comprehensive assessment frameworks, and targeted treatment approaches. In this spirit, Ami and I extend our deepest gratitude to the authors of these papers, the diligent reviewers whose contributions are integral to the journal’s output, the readers who engage with these works, the wider editorial staff, who made it all possible, and the journal managers for their thoughtful oversight.

Our greatest hope is that these efforts will inspire future research and foster connections within the academic community, empowering us to address loneliness in our work and personal lives.
